# Endogenous immunity at the forefront of tumor dormancy

**DOI:** 10.4155/fso.15.11

**Published:** 2015-11-01

**Authors:** Constantin N Baxevanis, Sonia A Perez

**Affiliations:** 1 Cancer Immunology & Immunotherapy Center, Saint Savas Cancer Hospital, 171 Alexandras avenue, Athens 11522, Greece

**Keywords:** cancer stem cells, endogenous immunity, immunoediting, immunotherapy, tumor dormancy

There is accumulating evidence to suggest that the efficacy of many of the currently used anticancer agents depends on the active contribution of patients’ endogenous immunity. Harnessing the immune system to achieve therapeutic efficacy is achievable via standard therapies as well as treatments designed to target oncogenic pathways in tumor cells. Immunomodulating antibodies by specifically blocking immune checkpoint inhibitors reinvigorate and potentially expand endogenous anticancer immune responses. Such responses can be also amplified by vaccines, which boost naturally occurring antitumor immune responses. The inherent capacity of the immune system to recognize tumor antigens and to control cancer cell growth has a major impact on the balance between dormant tumor cells and tumor escape. This piece will focus on the endogenous anticancer immunity as a basis for potential application to cancer immunotherapy strategies and to immune-mediated metastatic dormancy.

The immunoediting hypothesis, as proposed by R Schreiber [1], provided the platform based on which studies, later on, confirmed the role of endogenous antitumor immunity both as prognostic and predictive biomarkers. Thus, the presence, location and density of T cells and cytokines within tumors was shown to be related with a better prognosis, hence emphasizing the ability of the immune response to maintain a subclinical tumor in an equilibrium state [2]. Furthermore, such intratumoral immune signatures were shown to predict the outcome of chemotherapy or neoadjuvant therapy in various types of cancers [3,4].

The knowledge that endogenous immunity controls tumor growth and that tumor cells can use complex and overlapping mechanisms to avoid this immune detection has led investigators to target tumors through modulation of the immune response.

## Endogenous tumor immune surveillance as a major player for tumor dormancy

Presently, it is not known to which extent the immune composition of human tumors may influence the immunoediting process. Adaptive immune signatures intratumorally may reflect the fact that host antitumor immunological responses may protect the host from rapid tumor growth and thus prolong overall survival. Thus, immune surveillance will ‘edit’ the tumor during the late stages of immune surveillance at which time period the selective pressure mediated by the immune system has destroyed the immunogenic tumor variants leaving intact, or imposing the development of, less immunogenic ones [5]. The ‘edited’ tumor variants are predicted to proceed into a phase during which the immune system is continuously interacting with them, resulting in the establishment of a dynamic state of equilibrium. During equilibrium, tumor cells remain dormant for prolonged periods of time, lasting even for decades, in a process requiring active endogenous antitumor immunological responses mainly mediated by cytotoxic T lymphocytes. The outcome from the equilibrium phase depends on the balance between the strength and duration of the endogenous effector antitumor immunity and tolerance mechanisms developed by the tumor cells. Generally speaking, the equilibrium phase may end up in the elimination of all tumor cells, or may favor the selection for tumors which are no longer susceptible to immune attack, progressing into the phase of escape. Thus, the immunoediting theory proposes that endogenous immune mechanisms can induce and maintain tumor dormancy via a continuous durable pressure consisting of cytolytic and cytostatic functions whereby memory T cells act as the main players. The indispensable role of endogenous antitumor immunity for sustaining tumor dormancy or even eliminating dormant tumor cells is further supported by accumulating evidence suggesting that metastases may derive from very early disseminated tumor cells, even before the primary tumor becomes clinically detectable [6]. Such disseminated tumor cells are kept into dormancy or metastatic latency by host-derived cytotoxic T lymphocytes. It will be thus of instrumental importance to identify cellular and/or serum biomarkers which might help to detect dormant disease. In addition, transcriptional profiles from dormant disseminated tumor cells or experimental models of dormancy might help determine whether primary tumors carry a cancer dormancy ‘signature,’ which might have prognostic and also therapeutic value. It will be imperative to sustain such endogenous host-protective immune responses by booster immunizations during active immunotherapies [7,8] or by strategies reversing tumor-induced immune tolerance, such as targeted therapies with immune checkpoint inhibitors and kinase inhibitors [9] but also via conventional therapies [10], all of which could reinvigorate endogenous antitumor immunity and rise to memory cells.

## Role of the endogenous immunity within the tumor microenvironment in immune equilibrium

The prevalence of immune activation versus immune suppression within the tumor microenvironment has a key role in tipping the balance in favor of maintenance of tumor dormancy [11]. Many reports involving a wide variety of human cancers have indicated that infiltration of the tumor microenvironment by lymphocytes (tumor infiltrating lymphocytes; TIL) constitutes a robust prognostic indicator [12]. The identification of cancer types characterized by elevated infiltration of TIL has suggested that some patients may benefit from immune-based therapies. Recently, a large body of reports has revealed the importance of TIL in regulating the clinical progression of various epithelial cancers [2]. Nevertheless, the general consensus is that the dynamic relationship between effector and regulatory TIL may act either as an inauspicious or favorable prognostic factor having important consequences for overall survival [3,13]. Recent findings have indicated that the intratumoral effector immune profile in mice with dormant tumors was functionally active compared with a rather suppressed one in mice with progressing tumors, suggesting that high effector to suppressor cell ratios are associated with a constant equilibrium state and improved survival [6,14]. To this point it is worth mentioning that tumor dormancy also exists in disseminated tumor cells from early primary tumors [6,15] suggesting that dissemination occurs during, or even before, the process of immune surveillance. Metastatic latency in disseminated tumor cells also requires effector T lymphocytes, because their depletion accelerates tumor growth [6]. Thus, the status of immune-mediated dormancy in disseminated tumor cells during the growth of primary tumors introduces an additional equilibrium phase long before the end of immune surveillance [16]. Moreover, the existence of early disseminated tumor cells challenges the traditional view of acquisition of metastatic potential late during tumorigenesis. Genetic alterations favoring activation of genes promoting invasion and metastases surely distinguish the disseminated tumor cells from the rest in the primary tumor. Moreover, these cells should not be considered as ‘edited’ thus offering the chance for more effective immunotherapeutic modalities, based on a better understanding of the role of the immune system in metastatic dormancy.

## Cancer stem cells in clinical tumor dormancy

Tumor cells that survive during long dormancy periods should be resistant to immune attacks, and capable of switching from dormancy to malignancy. With regard to this, a puzzling issue should be how these cells avoid immune attacks either early disseminated from primary tumor, as nonedited, or after the phase of immune surveillance, as edited. One can easily postulate that this is a matter of lack of tumor immunogenicity, which makes the cells belonging to this subpopulation invisible by the immune effector cells. But, if this is indeed the case then why do these tumor cells not grow straight into overt tumors? A most likely explanation to answer this question would be to consider the existence of a poorly or nonimmunogenic subpopulation of tumor cells which after escaping immune attacks gives rise to a progeny of dormant cells, which initially are incapable to evolve to malignancy. There are several reports to suggest that cancer stem cells (CSCs) are often held responsible for tumorigenesis, tumor recurrences and metastases [17,18]. CSCs are long-lived, and can undergo multiple low-rate divisions giving rise to progeny consisting of variant clonal populations that can accumulate high number of genetic and epigenetic changes [17,19]. These variants may express neotumor antigens thus being susceptible to immune attacks. This process of immune-mediated elimination of genetically altered CSCs will apparently ensure latency prolongation because nonmutated CSCs (being nonimmunogenic and thus immune resistant) will be at low frequencies to grow fast and form clinically overt tumors. Notwithstanding, genomic instability in nonimmunogenic long-lived CSCs will provide a platform for accumulation of mutations favoring their activation and expansion [19]. Such activated CSCs will additionally employ mechanisms which either interfere with the generation of antitumor immune cells, thus reducing their numbers, or subvert them [20]. Consequently, gene sequencing analysis and protein profiling of unedited and edited disseminated tumor cells may reveal mutations involved in the process of exit from dormancy as a consequence of genomic instability. Specific mutations in these edited tumor cells may lead to altered expression of tumor-specific antigens for T cells, and these antigens could serve as immunotherapeutic targets.

## Future direction

Knowledge should be obtained on the conditions preserving immune dormancy and avoiding immune escape which may be the step in-between for developing modalities for eradicating disseminated dormant tumor cells. Malignant cells that escape the cytostatic or apoptotic effects induced by standard therapy will possibly enter a state of slow proliferation and dormancy, and will stay in this situation providing the treatment regimen is continuous. Conventional treatments are not promising approaches for prolonging tumor dormancy because dormant tumor cells are usually resistant to these therapies. Moreover, chemotherapy or radiotherapy lack specificity and memory, implying that their application should be continuous, something which is not feasible because of their toxicity against normal cells. It would be preferable if all treatments aiming at eradicating and restraining dormant tumor cells by potentiating endogenous antitumor immune mechanisms should be applied in patients in complete remission after surgical excision of their primary tumor, instead of doing this after the onset of metastatic disease. Moreover, when chemotherapy or radiotherapy is the initial treatment of patients after primary tumor excision, this should be combined with or immediately followed by immunotherapy in order to enable the immune system to put pressure over dormant disseminated tumor cells. The possibility of applying immunotherapeutic approaches at the ‘neoadjuvant’ setting should also be considered, since the resulting immune responses would have broader coverage of tumor variants also shared by disseminated dormant cells.
